# An accurate calculation method for inductor air gap length in high power DC–DC converters

**DOI:** 10.1038/s41598-024-54194-7

**Published:** 2024-02-12

**Authors:** Xiaohui Zeng, Wei Chen, Lei Yang, Qingbin Chen, Yuping Huang

**Affiliations:** 1 College of Electrical Engineering and Automation, Fuzhou University, Fuzhou, 350116 China; 2 Beijing Institute of Precision Mechatronics and Controls, Beijing, 100076 China

**Keywords:** Electrical and electronic engineering, Engineering

## Abstract

High-power inductors are fundamental components in high-power DC–DC converters, with their performance being a crucial metric of converter efficiency. This paper presents an in-depth analysis of a novel calculation method for the air gap length in such inductors. Taking into account the effects of air gap diffusion and the winding magnetic field, an expression for the air gap diffusion radius is derived, focusing on a distributed air gap structure. Furthermore, models for calculating the air gap and winding reluctance are developed, grounded in electromagnetic field theory. An equivalent magnetic circuit model, formulated based on Kirchhoff's second law, facilitates the proposed method for air gap length calculation. This study also involves the development of 3D models for both discrete and decoupled integrated inductors. The comparison between simulation outcomes and calculated air gap lengths indicates a maximum error of less than 8%, with the minimum error being as low as − 0.79%. Compared with traditional methods, the calculation method proposed in this paper has significant advantages. Additionally, the discrepancy between calculated values and experimental measurements is found to be 1.11%. These results validate the accuracy and applicability of the theoretical analysis and calculation method, underscoring their significance in the design and optimization of high-power DC–DC converters.

## Introduction

As power levels in power electronic devices escalate, the design requirements for magnetic components in DC–DC converters have intensified. Inductors, being pivotal in these converters, necessitate precise calculation of their parameters, a subject extensively discussed in existing literature^1–5^. A notable challenge in inductor design is the propensity of the magnetic core to saturate due to the substantial DC component present in inductors. To mitigate this, introducing a small air gap in the magnetic core's winding column has been identified as an effective strategy^6,7^. This approach becomes even more critical in high-power inductors, where larger air gaps are essential. Consequently, the accurate calculation of air gap length emerges as a key factor in the design of inductor parameters.

In standard practice, an air gap is incorporated into the winding column of the magnetic core. The length of this air gap can be determined by constructing a magnetic reluctance model, as delineated in (1). However, this method faces challenges, particularly in accurately determining the effective cross-sectional area of the air gap, which in turn affects the precision of the air gap length calculation using (1).

1 $$ l_{{\text{g}}} = \mu_{0} R_{{\text{g}}} A_{{\text{g}}} $$

In (1), *µ*_o_ represents the magnetic permeability of air, *R*_g_ is the magnetic reluctance of the air gap, and *A*_g_ is the effective cross-sectional area at the air gap.

Considering the diffusion effect of the air gap, the influence of the diffusion magnetic field depends on several factors, such as position, quantity, length, etc. Jez^8^ analyzed the impact of different air gap arrangements in the magnetic circuit on inductor parameters and proposed optimization criteria for distributed air gaps, which complicates the design process of inductors. Stenglein and Albach^9^ examined the air gap's size, shape, and position, indicating that these factors affect the effective cross-sectional area at the air gap. Subsequently, these factors are utilized to predict the size and position of the air gap.

Roshen^10^ analyzed the diffusion magnetic field of the air gap and proposed two methods to calculate the air gap diffusion magnetic field, which are more accurate when the air gap is relatively small. Maiti and Biswas^11^ and Yang^12^ introduced edge factors to explain the impact of air gap diffusion effects and redefined the effective cross-sectional area of the air gap. However, the effective cross-sectional area varies for different air gap models. Balakrishnan *et al*.^13^ proposed a method using the Schwartz-Christoff transform to calculate the magnetic reluctance of the air gap. However, this method is primarily applicable to situations where the air gap is small, and the analysis of the impact of air gap diffusion effects is not sufficiently deep. Kutkut and Divan^14^, Kuang *et al*.^15^ and Mukherjee et al.^16^ analyzed the influence of air gap size, air gap position, and thickness of the magnetic core between air gaps on winding losses. They also studied the air gap diffusion magnetic field under single, discrete, and uniform air gap distributions. Most existing literature focuses on cases where the air gap length is determined or in applications with small air gaps, analyzing the impact of air gap length on inductance, winding losses, and diffusion magnetic fields. However, calculating and designing the air gap length becomes challenging in situations with larger air gaps. Therefore, accurately calculating the air gap length for inductors is crucial in high-power DC–DC converters.

This paper comprehensively considers the air gap diffusion effect and equivalent winding reluctance and then establishes the air gap and winding reluctance models. The inductor's air gap diffusion reluctance and winding reluctance calculation methods are proposed based on a bidirectional DC–DC converter. According to the inductance value of the inductor in the DC–DC converter, the air gap length of each segment under the uniform distribution can be obtained. Finally, the correctness of this calculation method is verified by simulation and experiment.

The rest of the paper is organized as follows. The magnetic reluctance model of the discrete and decoupled integrated inductor is established in Magnetic reluctance analysis and modeling section”. The air gap, winding reluctance, and its calculation model are analyzed in “Winding reluctance analysis” section. The calculation method of the air gap length is proposed in “Air gap calculation” section. The theoretical analysis and calculation method are validated by simulations and experiments in “Simulation and experimental validation” section. Finally, some conclusions are drawn in “Conclusion” section.

## Magnetic reluctance analysis and modeling

**Figure 1:**
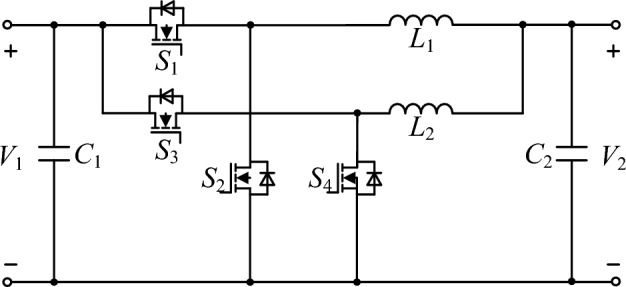
Bidirectional DC–DC converter circuit topology.

In high-power DC–DC converters, a significant DC component in inductors makes the magnetic core prone to saturation. Therefore, introducing an air gap becomes necessary to address this issue. For a pre-designed inductor in such a converter, parameters like inductance value, number of turns in the winding, magnetic core cross-sectional area, etc., are predetermined. The air gap length can be calculated by establishing the magnetic reluctance model of the inductor. This paper focuses on the topology of a high-power DC–DC converter, specifically an interleaved parallel Buck-Boost converter, with its circuit model depicted in Fig. 1. The technical specifications of the converter are detailed in Table 1, where the maximum forward power is 7.6 kW, and the maximum reverse power is 4.75 kW. Additionally, magnetic reluctance models for discrete inductors and decoupling integrated inductors have been established based on the structures of the two inductors in this topology.

**Table 1:** The specific technical specification of the converter.

Parameters	Parameter value
Capacitor voltage	200–400 V
DC bus voltage	130–190 V
Nominal DC bus voltage	160 V
Maximum forward current	40 A
Maximum forward power	7.6 kW
Forward discharge time	20 ms
Maximum reverse current	25 A
Maximum reverse power	4.75 kW
Reverse discharge time	30 ms
Inductance value	68 µH
Maximum inductor current	48 A
Maximum ripple current in the inductor	16 A
The effective current of the inductor	14.473 A
Switching frequency	100–300 kHz
Weight	< 2.5 kg

### Reluctance model of discrete inductor

**Figure 2:**
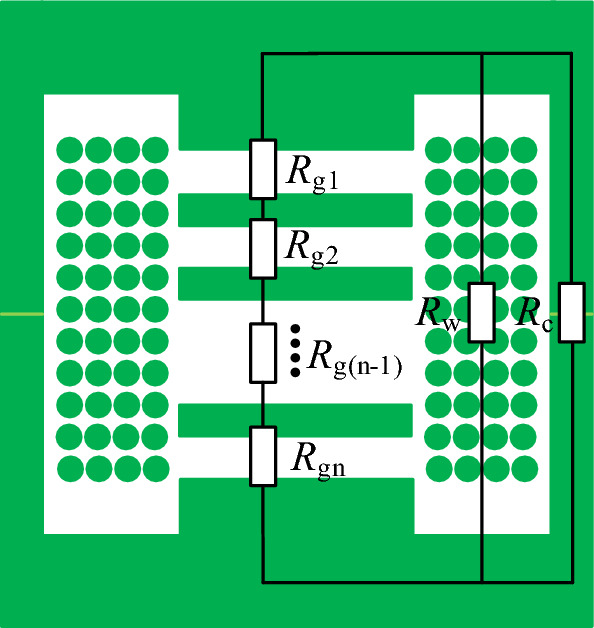
The reluctance model of the discrete inductor.

The magnetic core model for the discrete inductor is designated as PQ40/40. A reluctance model is established under a symmetric structure, considering only half of the magnetic core, as illustrated in Fig. 2. Employing a distributed air gap can meet the requirements for a larger air gap while reducing eddy current effects. In Fig. 2, *R*_g1_–*R*_gn_ denotes the reluctances of the first to nth segments of the air gap, and *R*_w_ stands for the equivalent winding reluctance of this model. For *R*_c_, which represents the magnetic reluctance of the magnetic core, its expression is as follows:

2 $$ R_{{\text{c}}} = \frac{{l_{{\text{c}}} }}{{\mu_{{\text{r}}} \mu_{{0}} A_{{\text{e}}} }} $$

where *l*_c_ is the magnetic path length, *µ*_r_ is the relative permeability, *A*_e_ is the cross-sectional area of the magnetic core winding column.

Since the magnetic core material is ferrite, its relative permeability is typically 3300. Therefore, *R*_c_ is much smaller than *R*_g_, and the magnetic reluctance of the core can be neglected. In the design of high-power inductors, compact and lightweight cores can be used to minimize the volume and weight of the transformer. Therefore, the number of turns in the winding should be increased to avoid core saturation. With the increase in the number of turns, the magnetic field intensity in the winding becomes non-negligible. Therefore, considering the winding reluctance when calculating the air gap length can enhance the accuracy of the calculations.

### Reluctance model of decoupled integrated inductor

**Figure 3:**
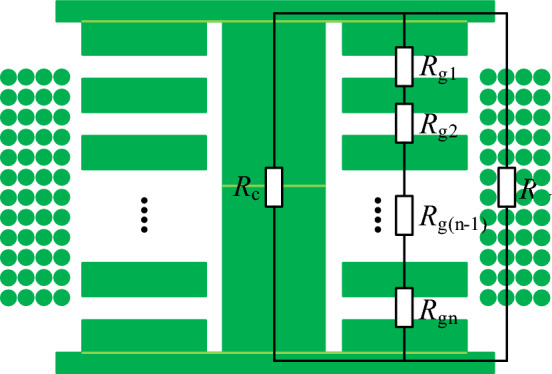
The reluctance model of the decoupled integrated inductor.

Building upon the reluctance model for the discrete inductor, we further derive the reluctance model for the decoupling integrated inductor, as shown in Fig. 3. The magnetic core for the decoupling integrated inductor also adopts a customized symmetric core structure.

### Reluctance calculation model

Based on the two inductor reluctance models established in the previous section, the following is a detailed analysis of the air gap and winding reluctance calculation methods under the reluctance model of discrete inductors.

### Air gap reluctance analysis

Inductors are components that store magnetic field energy. The high-permeability ferrite material studied in this paper cannot store significant energy. Therefore, the inductor magnetic core needs a low-permeability region to store energy. The simplest method is introducing an air gap into the ferrite magnetic core. Since the magnetic flux in the core can leak through the air gap, a significant diffusion magnetic flux is generated around the air gap, radiating a magnetic field outward. Therefore, the diffusion magnetic field of the air gap is significant. The magnetic field is closely related to magnetic reluctance, and the air gap reluctance can be divided into internal reluctance and diffusion reluctance.

**Figure 4:**
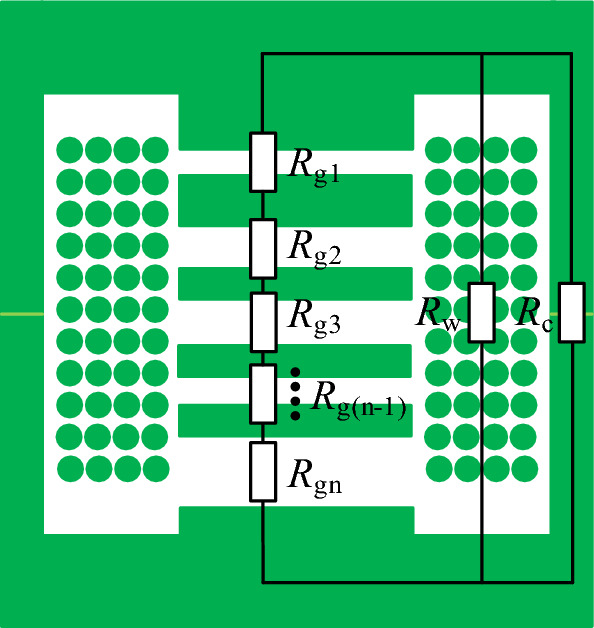
The reluctance model for non-uniform distributed gap.

In the gap calculation methods studied in this paper, there are primarily two approaches for modelling distributed gaps: uniform gap distribution and non-uniform gap distribution. When the gap is non-uniformly distributed, the magnetic reluctance model for discrete inductors evolves is shown in Fig. 4.

**Figure 5:**
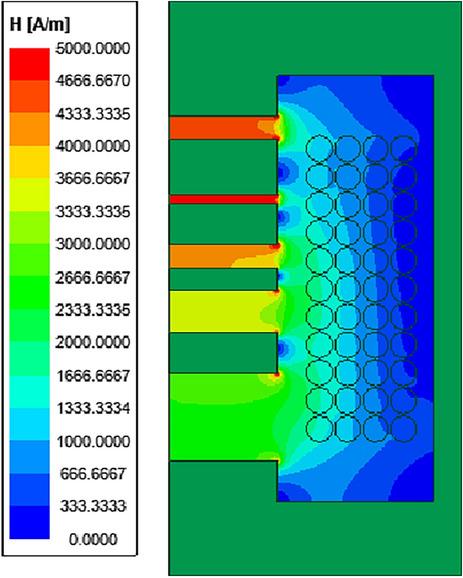
The distribution of the gap diffusing magnetic field under two-dimensional simulation.

Assuming the total length of the gap is 10 mm, the gap is divided into 5 segments. The distribution of the gap diffusing magnetic field under two-dimensional simulation is shown in Fig. 5.

From Fig. 5, it can be observed that the distribution of the diffusing magnetic field around the gap is irregular. Therefore, the selection and definition of each segment's gap length, the magnetic core column thickness between gaps, and the diffusing radius of the gap are challenging.

**Figure 6:**
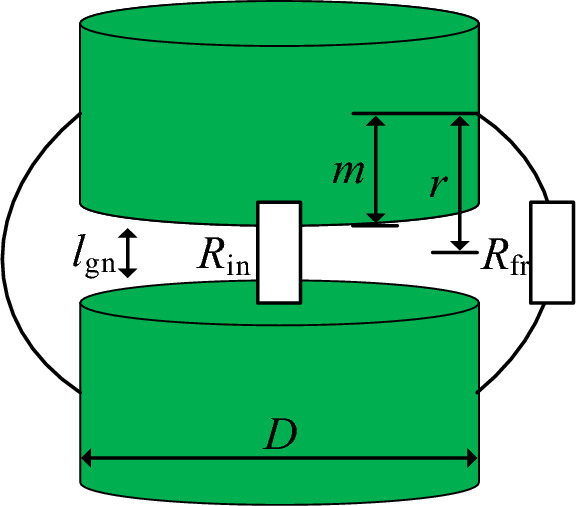
The model of air gap reluctance.

To achieve a more precise calculation of gap length, this paper employs a method of distributed gaps, where each segment of the gap is uniformly distributed. In addition, the thickness of the core between the air gaps is also evenly distributed. The model of the nth segment of the air gap is shown in Fig. 6.

In Fig. 6, *R*_in_ represents the inner reluctance of the air gap. *R*_fr_ is the air gap diffusion reluctance. *r* is the diffusion radius of the air gap. *l*_gn_ is the air gap length in the nth segment. *D* is the diameter of the magnetic core winding column. *m* is defined as half of the thickness of the magnetic core column between air gaps, and its expression can be obtained in (3).

3 $$ m = \frac{{\frac{{h - n \cdot l_{{{\text{gn}}}} }}{n + 1}}}{2} $$

**Figure 7:**
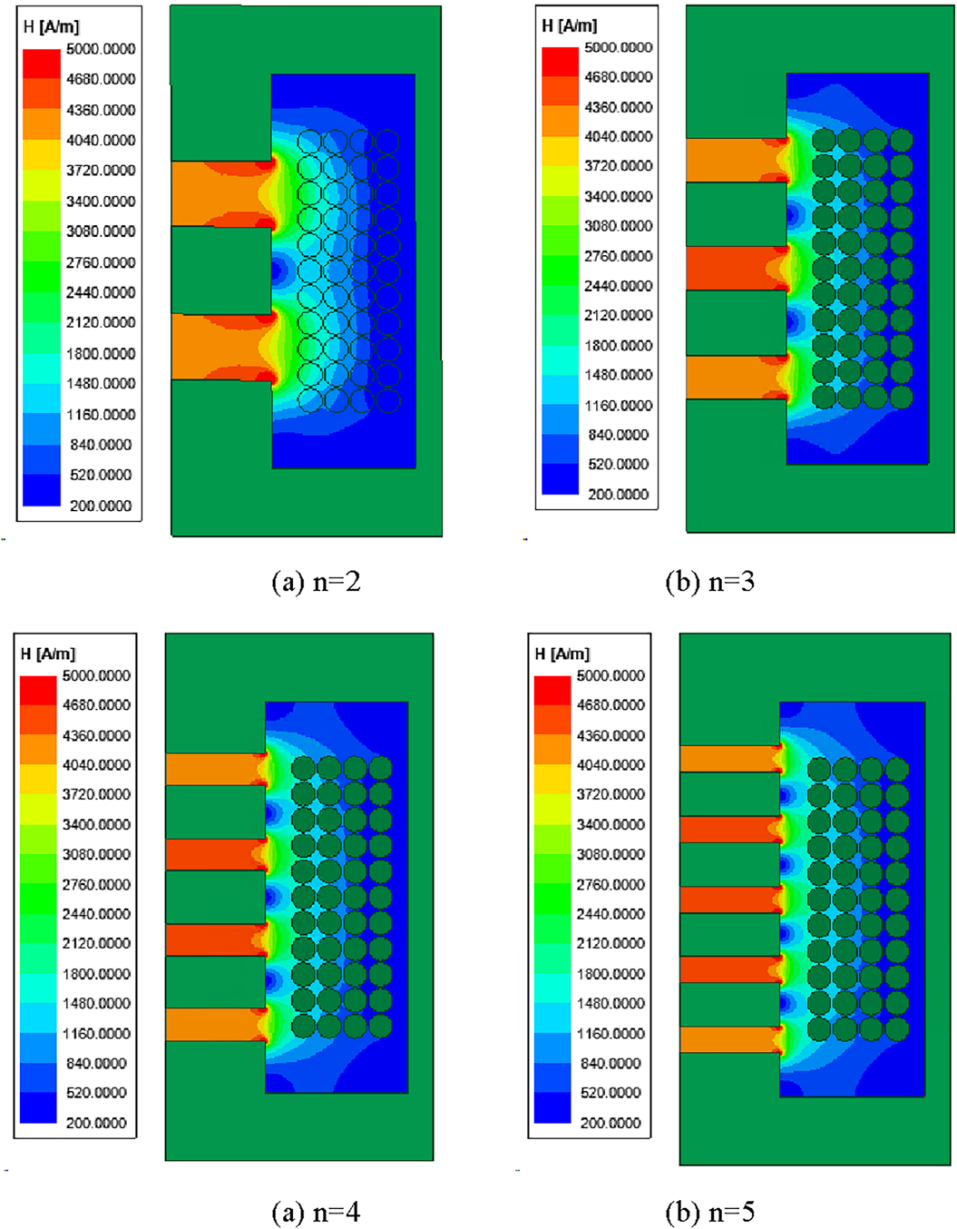
The 2D magnetic field distribution for different numbers of segments when the air gap length is 10 mm.

For the selection of the air gap diffusion radius, an initial analysis was conducted on the distribution of the magnetic field around the air gap. When the air gap length is fixed, dividing the air gap into different segments results in varying diffusion magnetic field ranges around the air gap. Figure 7 depicts the 2D magnetic field distribution for different numbers of segments when the air gap length is 10 mm. It can be observed that as the number of segments increases, the diffusion radius of the magnetic field around the air gap decreases. Furthermore, it is deduced that the diffusion magnetic field range follows a certain pattern: half of the air gap length plus half of the magnetic core length between the gaps.

From Fig. 7, it can be observed that the diffusion radius of the magnetic field around the air gap can be defined as shown in (4). The magnetic reluctance of the nth segment of the air gap can be equivalent to the sum of the internal magnetic reluctance of the air gap and the diffusion magnetic reluctance around the air gap, as shown in (5).

4 $$ r = m + \frac{{l_{{{\text{gn}}}} }}{2} $$

5 $$ R_{{{\text{gn}}}} = R_{{{\text{in}}}} + R_{{{\text{fr}}}} $$

where *h* is the height of the magnetic core winding column, and n is the number of air gaps.

For a cylindrical end-face magnetic core, assuming that the magnetic field in the air gap is vertical and uniformly distributed inside the air gap, the calculation of the magnetic conductivity within the gap is related to *l*_gn_/*D*. According to practical engineering experience, when *l*_gn_/*D* is less than 0.3, the formula for calculating the magnetic conductivity within the gap can follow the conventional gap calculation method.

6 $$ G_{{{\text{in}}}} = \frac{{\mu_{0} A_{{\text{e}}} }}{{l_{{{\text{gn}}}} }} = \frac{{\mu_{0} D^{2} }}{{4l_{{{\text{gn}}}} }} $$

where *A*_e_ is the cross-sectional area of the magnetic core winding column.

Since the calculation method in this paper is based on distributed gaps, the size of each gap segment varies when the gap is divided into different sections. Table 2 provides the ratio of air gap size to the core diameter under different numbers of air gap sections.

**Table 2:** The ratio of gap size to the ratio of air gap size to the core diameter (*l*_gn_/*D*).

n	*l*_gn_/*D*
1	0.952
2	0.487
3	0.266
4	0.192
5	0.153
6	0.13
7	0.113

To maintain consistency, this paper considers only the case where *l*_gn_/*D* is less than 0.3 in the calculation of the magnetic conductivity within the gap.

**Figure 8:**
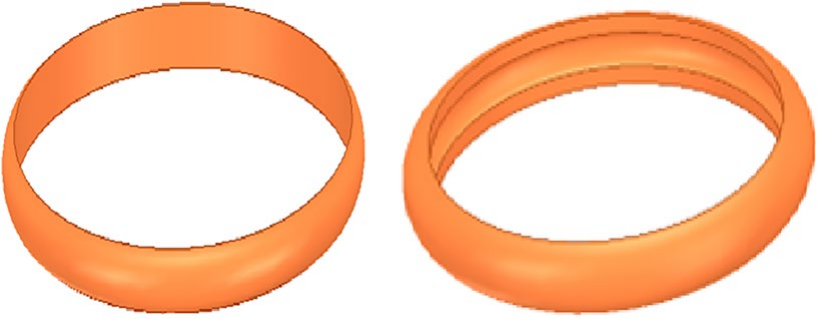
The model of the annulus and annulus shell.

In most cases, the magnetic flux passes not only through the surface of the magnetic core but also through the edges, corners, and the side surface of the magnetic core near the air gap. The magnetic core winding column studied in this paper is cylindrical. At the same time, analyzing the size of the diffusion magnetic flux and the diffusion radius in actual situations is quite complex. Therefore, this paper employs the magnetic field segmentation method for analysis, which is based on estimating potential flux paths. It divides the magnetic field into several magnetic flux tubes with regular shapes, then uses an analytical approach to calculate the magnetic conductivity of these flux tubes. Finally, the total diffusing magnetic conductivity of the gap is obtained. Regarding the cylindrical magnetic core model adopted in this paper, the diffusion magnetic flux path is divided into two parts^17^: the annulus and the annular shell, as shown in Fig. 8.

**Figure 9:**
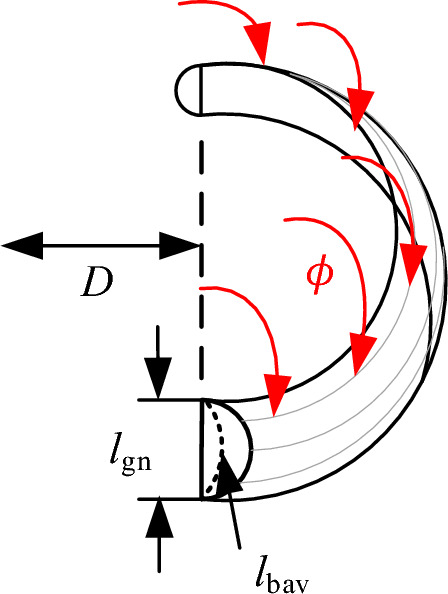
The magnetic conductivity calculation model for the annular.

The magnetic conductivity calculation model for the annular section is illustrated in Fig. 9.

The expression for the magnetic conductivity of the annular gap section in Fig. 9 is as follows:

7 $$ G_{{{\text{fr1}}}} = \frac{{\mu_{0} A_{{{\text{bav1}}}} }}{{l_{{{\text{bav1}}}} }} $$

where *A*_bav1_ is the average cross-sectional area in the direction indicated by the arrow in Fig. 9. *l*_bav1_ is the average magnetic path length for this section, as indicated by the dashed line in Fig. 9. The size of *l*_bav1_, determined by the actual plotting method, is given by the following:

8 $$ l_{{{\text{bav1}}}} = 1.22l_{{{\text{gn}}}} $$

To calculate the effective cross-sectional area of the annular, the expression for the effective volume of the annular is as follows:

9 $$ V_{{{\text{bav1}}}} = \frac{{2\pi \left( {\frac{D}{2} + \frac{{l_{{{\text{gn}}}} }}{2}} \right)\frac{\pi }{2}\left( {\frac{{l_{{{\text{gn}}}} }}{2}} \right)^{2} }}{{l_{{{\text{bav1}}}} }} $$

Therefore, the expression for the effective cross-sectional area of the annular ring is shown in (10).

10 $$ A_{{{\text{bav1}}}} = \frac{{V_{{{\text{bav1}}}} }}{{l_{{{\text{bav1}}}} }} $$

Finally, the expression for the magnetic conductivity of the annular gap section can be simplified as follows:

11 $$ G_{{{\text{fr1}}}} = 1.63\mu_{0} \left( {\frac{{D + l_{{{\text{gn}}}} }}{2}} \right) $$

**Figure 10:**
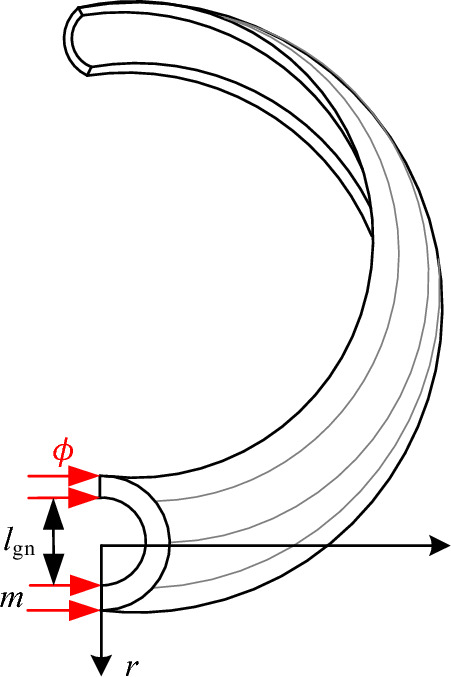
The magnetic conductivity calculation model for the annular shell.

Based on the above analysis method, the gap magnetic conductivity calculation model for the annular shell section is illustrated in Fig. 10.

By integrating the magnetic field strength over the annular shell, the expression for the gap magnetic conductivity is derived as follows:

12 $$ \begin{aligned} G_{{{\text{fr2}}}} & = \frac{{\phi_{{{\text{bav2}}}} }}{{N_{{\text{p}}} I}} = \mu_{0} \frac{{A_{{{\text{bav2}}}} }}{{N_{{\text{p}}} I}}\int {Hdr} \\ & = \mu_{0} \frac{{A_{{{\text{bav2}}}} }}{{N_{{\text{p}}} I}}\int {\frac{{N_{{\text{p}}} I}}{{l_{{{\text{bav2}}}} }}dr} \\ \end{aligned} $$

Further, the expression for the gap magnetic conductivity of the annular shell can be obtained as follows:

13 $$ \begin{aligned} G_{{{\text{fr2}}}} & = \mu_{0} \int_{{\frac{{l_{{{\text{gn}}}} }}{2}}}^{{\frac{{l_{{{\text{gn}}}} }}{2} + {\text{m}}}} {\frac{{2\pi \left( {\frac{D}{2} + \frac{{l_{{{\text{gn}}}} }}{2}} \right)}}{\pi r}dr} \\ & = \mu_{0} (D + l_{{{\text{gn}}}} ){\text{ln}}\left( {{1 + }\frac{2m}{{l_{{{\text{gn}}}} }}} \right) \\ \end{aligned} $$

Finally, the reluctance expression of the nth air gap can be derived by combining (6), (11), (13) as

14 $$ \begin{aligned} R_{{{\text{gn}}}} & = \frac{1}{{G_{{{\text{in}}}} + G_{{{\text{fr1}}}} + G_{{{\text{fr2}}}} }} \\ & = \frac{1}{{\mu_{0} \left( {\frac{{A_{{\text{e}}} }}{{l_{{{\text{gn}}}} }} + 1.63\left( {\frac{{D + l_{{{\text{gn}}}} }}{2}} \right) + (D + l_{{{\text{gn}}}} )\ln \left( {1 + \frac{2m}{{l_{{{\text{gn}}}} }}} \right)} \right)}} \\ \end{aligned} $$

## Winding reluctance analysis

In this section, the reluctance model of the discrete inductor is chosen to calculate the winding reluctance. When the number of turns and the current in the winding are determined, the magnitude of the magnetic flux in the entire winding section can be obtained. Subsequently, the expression for the winding reluctance can be obtained, as shown in (15).

15 $$ R_{{\text{w}}} = \frac{{\psi_{{\text{w}}} }}{{N^{2} I}} $$

**Figure 11:**
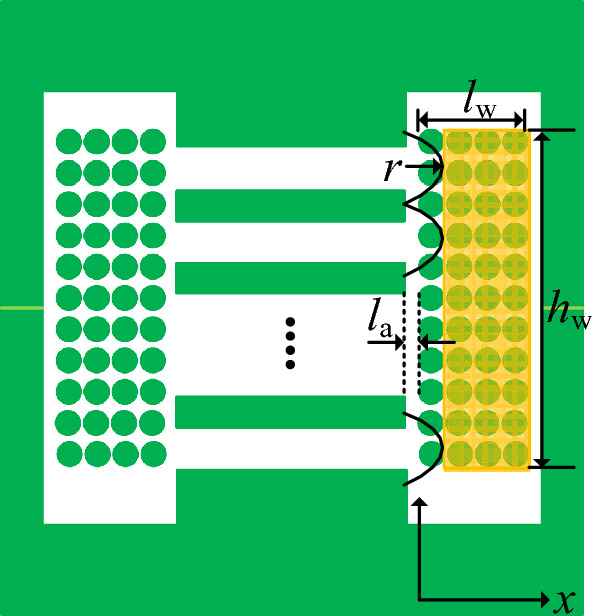
The calculation model of the winding flux link.

This paper will adopt a calculation method similar to that used for calculating transformer leakage inductance to calculate the winding magnetic flux accurately^18^. When the magnetic flux inside the winding is uniformly and parallelly distributed, the calculation model is shown in Fig. 11. Since the diffusion reluctance of the air gap has been considered when calculating the air gap reluctance, the influence of the diffusion magnetic field of the air gap can now be ignored.

In Fig. 11, by calculating the magnetic flux in the yellow area and then deriving the expression for the total magnetic flux as follows:

16 $$ \begin{aligned} \psi_{{\text{w}}} & = \frac{x}{{l_{{\text{w}}} }}N\int_{0}^{{l_{{\text{w}}} + l_{{\text{a}}} - r}} {\phi dx} \\ & = \frac{x}{{l_{{\text{w}}} }}N\int_{0}^{{l_{{\text{w}}} + l_{{\text{a}}} - r}} {\frac{{\frac{x}{{l_{{\text{w}}} }}NI}}{{\frac{{H_{{\text{w}}} }}{{\mu_{0} MLT}}}}dx} \\ & = \frac{{\mu_{0} N^{2} IMLT(l_{{\text{w}}} + l_{{\text{a}}} - r)^{3} }}{{3l_{{\text{w}}}^{2} H_{{\text{w}}} }} \\ \end{aligned} $$

where: *MLT* is the average turn length of the winding. *H*_w_ is the equivalent magnetic circuit length of the winding. The Rogowski factor is introduced to calculate the equal winding height in this section, and its expression is shown in (17)^19^.

17 $$ K = 1 - \frac{{1 - e^{{ - \frac{{\pi h_{{\text{w}}} }}{{l_{{\text{w}}} }}}} }}{{\frac{{\pi h_{{\text{w}}} }}{{l_{{\text{w}}} }}}} $$

According to the Rogowski factor, the equivalent winding height is represented as.

18 $$ H_{{\text{w}}} = \frac{{h_{{\text{w}}} }}{K} $$

where *h*_w_ is the actual winding height.

Combining (15), (16) and (18), the expression for the winding reluctance is as follows:

19 $$ R_{{\text{w}}} = \frac{{N^{2} I}}{{\psi_{{\text{w}}} }} = \frac{{3l_{{\text{w}}}^{2} H_{{\text{w}}} }}{{\mu_{0} MLT(l_{{\text{w}}} + l_{{\text{a}}} - r)^{3} }} $$

## Air gap calculation

**Figure 12:**
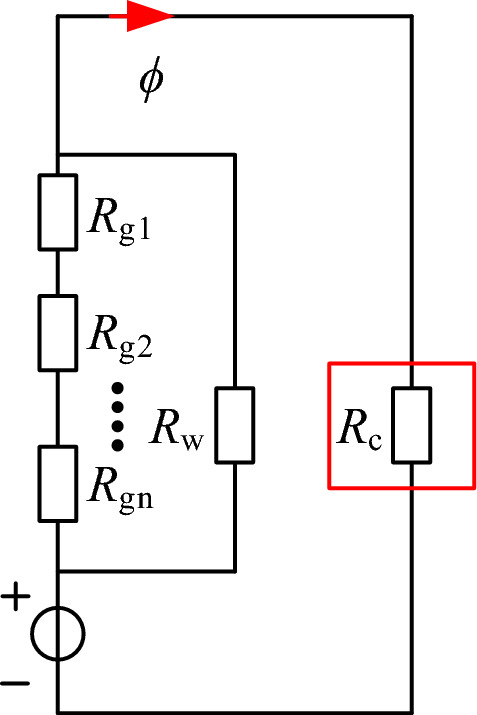
Equivalent magnetic circuit model.

Based on the calculated formulas for air gap reluctance and winding reluctance from the previous analysis, an equivalent magnetic circuit for the inductor needs to be established to determine the length of each air gap. Assuming a uniform distribution of air gaps and equal size for each segment of the air gap. Neglecting the reluctance of the magnetic core, the equivalent magnetic circuit model for the discrete inductor is shown in Fig. 12.

According to Kirchhoff's second law of magnetic circuits, the expression for the inductance can be derived as

20 $$ L = \frac{{N^{2} }}{{\left( {\sum\limits_{i = 1}^{n} {R_{{{\text{gi}}}} } } \right){//}R_{{\text{w}}} }} = \frac{{N^{2} }}{{\left( {nR_{{{\text{gn}}}} } \right){//}R_{{\text{w}}} }} $$

When the inductance value in the DC–DC converter is known, a theoretical air gap size can be calculated based on (1). However, for large air gap inductors, the air gap length calculated by (1) differs significantly from the actual length. Therefore, to accurately calculate the required air gap size, this paper, based on the established models for air gap reluctance and winding reluctance, further simplifies (20) to the following:

21 $$ L = \frac{{N^{2} }}{{\left[ {(nR_{{{\text{gn}}}} ){//}\frac{{3l_{{\text{w}}}^{2} H_{{\text{w}}} }}{{\mu_{0} MLT(l_{{\text{w}}} + l_{{\text{a}}} - r)^{3} }}} \right]}} $$

In (21), all parameters except the air gap size are determined, so the air gap size can be solved.

## Simulation and experimental validation

**Figure 13:**
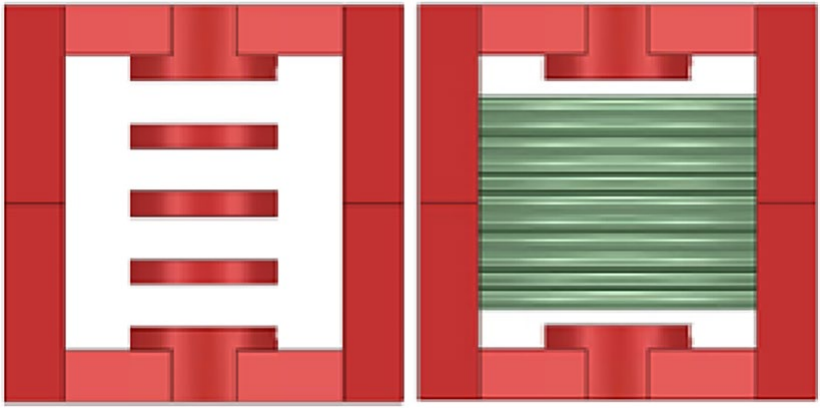
3D model of the discrete inductor.

**Figure 14:**
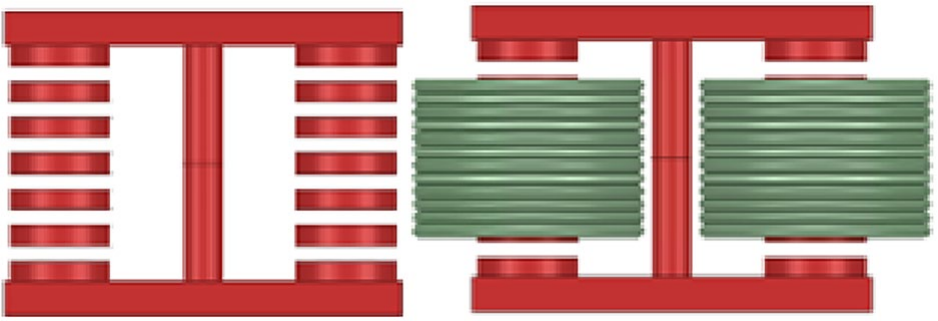
3D model of the decoupled integrated inductor.

The 3D simulation models for the discrete inductor and the decoupled integrated inductor are shown in Figs. 13 and 14. The accuracy of the air gap length calculation method is verified through the simulation of the two inductor models. When the inductance value is known, the size of each segment of the air gap under different analyses is calculated and compared with the simulation results. Additionally, the method is validated for different inductance values. Finally, the error of this calculation method is obtained, confirming the correctness of the calculation method.

### Air gap verification of discrete inductor

Table 3 provides the electrical parameters of discrete inductors, which are crucial for the design of the inductor, including the magnetic core cross-sectional area and the number of turns in the winding. This paper specifically focuses on analyzing the gap size in the design of the inductor, while other design results are directly presented in this paper. Next, the results for the number of gap sections (n) ranging from 2 to 7 were analyzed, as this range is commonly used in practical engineering applications. Furthermore, the comparison between the results of the proposed calculation method, the maxwell simulation and traditional method under different segments are shown in Fig. 11.

**Table 3:** The parameters of the discrete inductor.

Parameters	Value
Ae (mm^2^)	183.6
*L* (µH)	68
*N*	44
*MLT* (mm)	84
*D* (mm)	15.04
*l*_a_ (mm)	2
*l*_w_ (mm)	7.7
*h*_w_ (mm)	21.35
*h* (mm)	29.7

Concerning the discrete inductor model in the paper, simulation results reveal that the required gap length to achieve the target inductance is 14.32 mm, while the actual diameter of the magnetic core only 15.04 mm. The ratio of the air gap size to the core diameter has reached 0.952. This will impact the calculation of the gap diffusion radius. Using the method proposed in this paper, the calculated required gap length is 50.705 mm. The gap length has exceeded the height of the magnetic core window, which is not suitable. Therefore, the focus of the calculation method proposed in this paper is based on the scenario of distributed gaps, with at least two or more gap sections. This is also the reason why the case of n = 1 is not considered.

**Figure 15:**
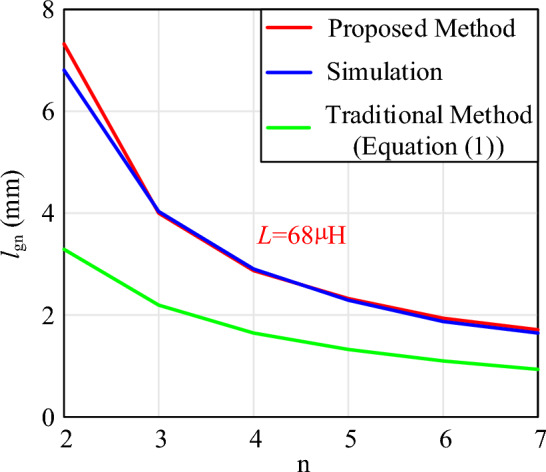
The results of the proposed method, simulation results of different segments (*L* = 68 µH).

Furthermore, the comparison between the results of the proposed calculation method, the Maxwell simulation and the traditional method under different segments are shown in Fig. 15.

**Figure 16:**
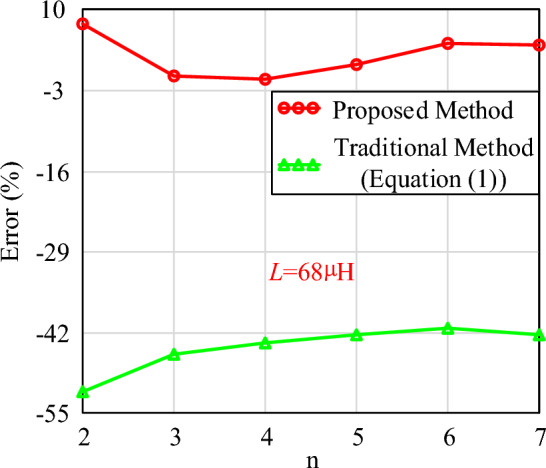
The calculation errors (*L* = 68 µH).

Figure 15 shows the variation of the calculated and simulated air gap results with n when the inductance value is known. As the number of air gap segments increases, the length of each segment decreases. Meanwhile, the calculation errors at different numbers of air gap segments are shown in Fig. 16. The results indicate a good agreement between the proposed calculated and simulated results, demonstrating the high accuracy of the proposed calculation method. The maximum error is 7.61%, and the minimum error is − 0.79%. The reason for this large error is also due to the fact that when the air gap is divided into two sections, the ratio of air gap size to core length is greater than 0.3. Therefore, the limiting condition proposed in this paper is also to ensure that this ratio is less than 0.3. However, compared to traditional methods, the calculation method proposed in this paper has distinct advantages.

**Figure 17:**
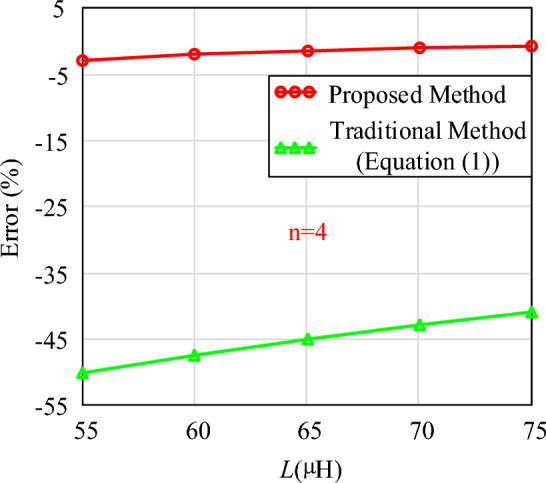
The calculation errors of different inductance value (n = 4).

**Figure 18:**
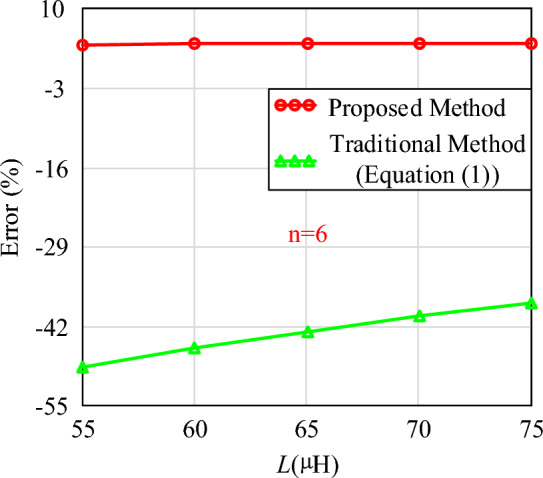
The calculation errors of different inductance value (n = 6).

To validate the flexibility of this calculation method, Figs. 17 and 18 show the required air gap lengths for different inductance values when n = 4 and n = 6, respectively.

In Figs. 17 and 18, the calculation errors of the proposed method are all less than 5%. Compared with traditional methods, the calculation method proposed in this paper also exhibits significant advantages under different target inductance values. Therefore, whether the inductance value is constant or variable, the method introduced in this paper can calculate the required air gap size. It can also calculate the air gap size for each segment in the case of distributed air gaps.

### Air gap verification of decoupled integrated inductor

The calculation method proposed in this paper also applies to the decoupled integrated inductor model analyzed earlier. Table 4 presents the electrical parameters of the decoupled integrated inductor. According to the requirements of the DC–DC converter specifications, the air gap length calculated using this method is in good agreement with the simulation results when the inductance value is 68 µH.

**Table 4:** The parameters of the decoupled integrated inductor.

Parameters	Value
*A*_e_ (mm^2^)	181
*L* (µH)	68
*N*	48 turns
*MLT* (mm)	84.5 mm
*D* (mm)	15.2 mm
*l*_a_ (mm)	2 mm
*l*_w_ (mm)	7.7 mm
*h*_w_ (mm)	23.3 mm
*h* (mm)	35

**Figure 19:**
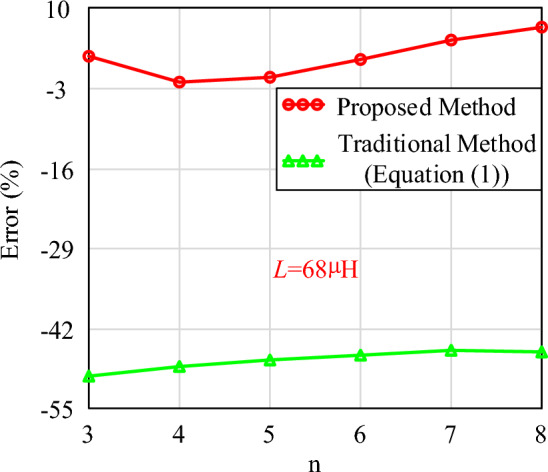
The calculation errors (*L* = 68 µH).

When the inductance value is 68 µH, the calculation errors at different numbers of air gap segments are shown in Fig. 19. The maximum error of this method is 6.79%, and the minimum error is − 1.19%. This further validates the correctness of the calculation method.

### Experimental validation

**Figure 20:**
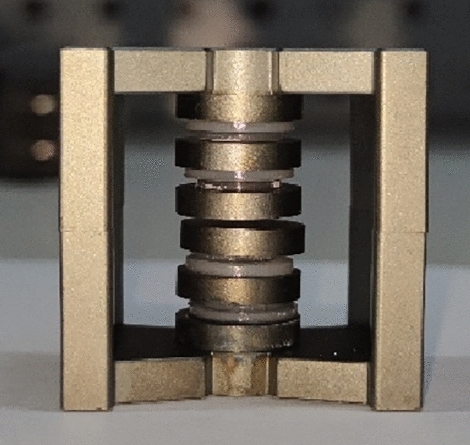
The inductor without windings.

**Figure 21:**
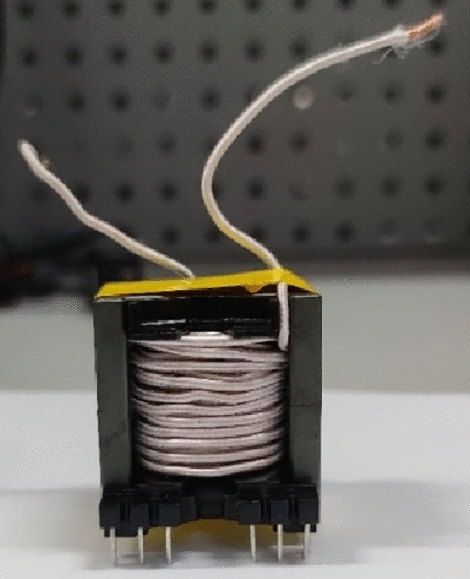
The inductor with windings.

This section constructs a discrete inductor sample to verify the correctness of the calculation method. The target inductance value for this discrete inductor is 68 µH. Under the condition of n = 5, the air gap size can be calculated using the formula proposed in this paper. The calculated length of the air gap for the nth segment *l*_gn_ is 2.307 mm. However, there is a certain error in the sample's air gap length. Five insulating pads replace the air gap with a thickness of 2.3 mm each. Finally, the parameters of the experimental sample are *L* = 68 µH, *N* = 44, *n* = 5, *l*_gn_ = 2.3 mm. Figure 20 shows the inductor without windings, and Fig. 21 shows the inductor with windings.

**Figure 22:**
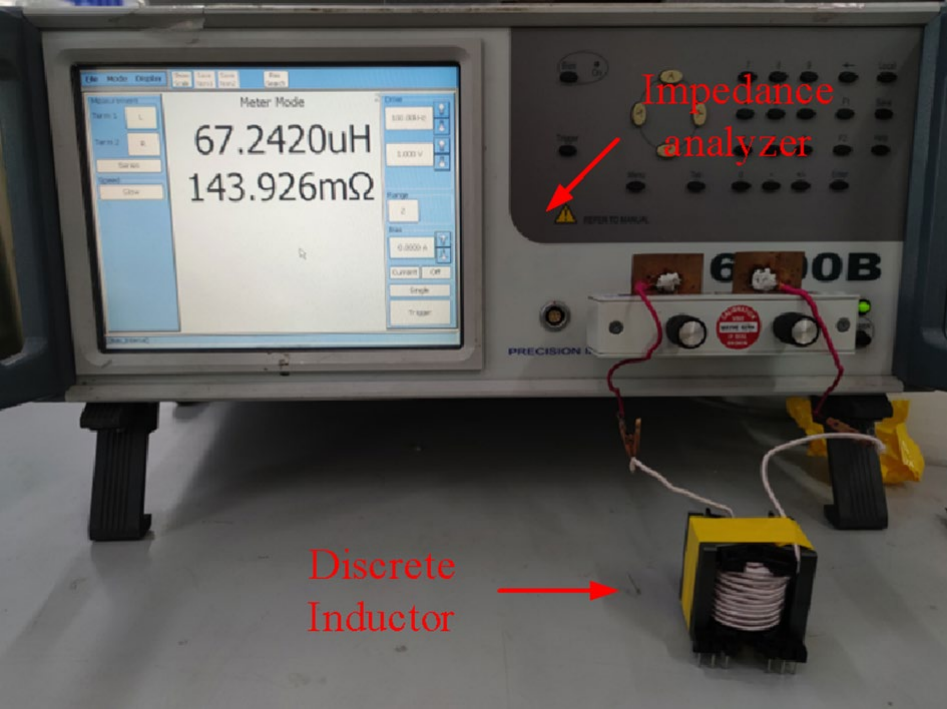
The experimental platform.

The experimental platform is shown in Fig. 22, which includes a precision impedance analyzer and the inductor sample. The frequency and bias current of the device during the measurement of inductance values are 100 kHz and 0 A, respectively.

Table 5 shows that the experimentally measured inductance value is 67.24 µH, while the calculated inductance value using this method is 68 µH. The experimental results indicate that the calculation error of this method is − 1.11%.

**Table 5:** The comparison of *L.*

Parameters	Theoretical value	Simulated value	Measured value
*L* (µH)	68	67.851	67.24

## Conclusion

This paper delves into the application of the reluctance model for accurately calculating the air gap in inductors, particularly under conditions of a large air gap, using a distributed air gap approach. The focus is on discrete and decoupled integrated inductors within the context of bidirectional two-channel DC–DC converters. By employing electromagnetic field theory, models for both air gap and winding reluctance are established. This includes a division of air gap reluctance into inner and diffusion components, with a specific emphasis on defining the radius of the air gap diffusion effect, a critical factor in determining air gap length. In the process of calculating winding reluctance, the air gap diffusion field is notably omitted, and the Rogowski factor is introduced to ascertain the equivalent winding height. Additionally, the paper posits that air gap diffusion reluctance can be equated to both ring and ring shell forms, highlighting its significance in influencing the overall air gap diffusion reluctance and the precision of air gap length calculations. Comparative analysis of calculated air gap lengths, based on both discrete and decoupled integrated inductors, against simulated and measured results, is presented. The findings indicate that the discrepancy between calculated and simulated values is less than 8%, with a minimum error margin of − 0.79%, under both constant and variable inductance conditions. Compared with traditional methods, the calculation method proposed in this paper has significant advantages. The error margin between calculated and measured values stands at − 1.11%. The concluding part of the paper confirms the validity and utility of the theoretical framework and the proposed calculation method through simulation and experimental results.

## Data Availability

The authors declare that the data supporting the findings of this study are available within the article and its supplementary information files. All other relevant data are available from the corresponding author upon reasonable request.
